# Impact of Sex on Age-Related Changes in Pancreatic Islet Morphology and Beta-Cell Plasticity in Mice

**DOI:** 10.3390/ijms27198479

**Published:** 2026-09-23

**Authors:** Vaibhav Dubey, Andrei I. Tarasov, Neil Tanday, Peter R. Flatt, R. Charlotte Moffett, Nigel Irwin

**Affiliations:** Diabetes Research Centre, Schools of Biomedical Sciences and Pharmacy & Pharmaceutical Sciences, Ulster University, Coleraine BT52 1SA, UK; v.dubey@ulster.ac.uk (V.D.); a.tarasov@ulster.ac.uk (A.I.T.); n.tanday@ulster.ac.uk (N.T.); pr.flatt@ulster.ac.uk (P.R.F.); cr.moffett@ulster.ac.uk (R.C.M.)

**Keywords:** ageing, islet, cell lineage, sex, transdifferentiation

## Abstract

The aim of the current study was to assess the impact of age and sex on pancreatic islet morphology and related islet cell lineage. To accomplish this, virgin male and female transgenic Ins1*^Cre/+^*;Rosa26-eYFP and Glu*^CreERT2^*;ROSA26-eYFP mice were employed at 12 and 52 weeks of age, with sex- and age-dependent effects on pancreatic islet structure, and particularly the influence of changes in islet cell plasticity, examined. At 12 weeks of age, male Ins1*^Cre/+^*;Rosa26-eYFP mice exhibited increases in overall pancreatic islet area and number, as well as alpha- and beta-cell areas when compared to similarly aged females. This was associated with reduced beta-cell apoptosis and a preservation of beta-cell identity, alongside a prominent increase in the association of ductal and beta-cell markers. However, at 52 weeks of age, there was an almost complete sex-specific reversal of effects on islet morphology. Older female mice presented with enhanced islet- and beta-cell area when compared to respective males, which was accompanied by substantially reduced beta-cell apoptosis rates, up-regulated beta-cell neogenesis, plus markedly reduced beta-cell dedifferentiation. Observations in related Glu*^CreERT2^*;ROSA26-eYFP transgenic mice further highlighted the promotion of islet cell transitioning events in female mice that encourage preservation of beta-cell mass in response to ageing. In conclusion, there are clear sex- and age-provoked differences in pancreatic islet morphology. Such changes are partly linked to modification of islet cell turnover rates, and especially the ability of female, but not male, mice to effectively modify islet cell plasticity towards retention of beta-cell mass in the face of age-induced stress.

## 1. Introduction

Sex represents an underappreciated biological variable within both preclinical and clinical research environments, despite disparities in metabolic status between males and females [1]. Accordingly, there is evidence of sex-related differences in glucose homeostasis as well as responses to established anti-hyperglycaemic drugs [2,3], with males possessing a greater risk of developing diabetes and related metabolic disorders than females [4]. A network meta-analysis confirms age-related differences in efficacy and patient outcomes with newer treatment options for type 2 diabetes and suggests the possibility of sex-related variations [5]. There is also evidence that female islets exhibit prolonged graft survival following transplantation [6]. In keeping with this, sexual dimorphism is apparent in terms of pancreatic islet morphology, which is believed to be influenced by both hormonal and genetic factors [7]. It follows that the main sex hormones, testosterone and oestrogen, directly impact islet structure, with oestrogen generally considered to exert protective islet cell actions [8], whereas the exact influence of testosterone is still controversial [9]. Indeed, the aforementioned metabolic advantages in females are absent following the onset of menopause [10].

Regardless, there are still many unknowns regarding the sex-specific impact of ageing on pancreatic islet morphology. The ageing process is a long-recognised islet cell stressor, typically linked to the development of insulin resistance and impaired glucose tolerance [11]. Ultimately, this leads to an enhanced metabolic workload for insulin-secreting beta cells and resultant adaptive changes in pancreatic islet cell composition and morphology, most notably exhaustion of functional beta-cell mass [12]. Whilst stress-induced elevations of beta-cell apoptosis are likely to be involved in this process, observations of minimal changes in beta-cell survival rates between young and old individuals suggest alternative mechanisms must be at play [13]. Consequently, one such process that has received much recent attention in this respect is the influence of deleterious changes in pancreatic islet cell lineage [14]. Using transgenic mice, we have recently demonstrated that disturbed pancreatic islet morphology and beta-cell function in middle-aged male mice is linked to loss of beta-cell identity alongside reduced transdifferentiation of alpha and ductal cells towards a beta-cell phenotype [15].

In the present study, we have employed male and female mice from the same transgenic mouse colony, namely Ins1*^Cre/+^*;Rosa26-eYFP, at 12 and 52 weeks of age to investigate sex-dependent effects of ageing on pancreatic islet cell plasticity [15,16,17], particularly beta-cell lineage and related beta-to-alpha-cell transdifferentiation events. Equivalently, middle-aged male and female transgenic Glu*^CreERT2^*;ROSA26-eYFP mice with islet alpha-cell lineage tracing capabilities were also used to specifically investigate corresponding changes in alpha-to-beta-cell lineage. Importantly, the Glu*^CreERT2^*;ROSA26-eYFP model is already associated with sex-specific differences in pancreatic islet morphology adaptations to various independent metabolic stressors [18], and in keeping with this, ageing has been established to impart detrimental changes on islet cell plasticity in male mice [15]. The current study extends these initial observations and offers distinct novelty by being the first to directly compare the impact of sex on age-related deterioration of islet cell transdifferentiation events and their relationship to the overall integrity of pancreatic islet cell morphology.

## 2. Results

### 2.1. Male Mice Have Increased Body Weight and Age-Dependent Elevation of Circulating Glucose

Younger male Ins1*^Cre/+^*;Rosa26-eYFP transgenic mice had elevated (*p* < 0.01) body weight when compared to respective females, but non-fasted circulating glucose levels were similar in both sexes at 12 weeks of age (Table 1). Increased (*p* < 0.01) body weight was still apparent at 52 weeks of age in male mice, but by this point these mice had significantly greater (*p* < 0.01) blood glucose levels than corresponding females (Table 1).

### 2.2. Male Mice Exhibit an Age-Dependent Decline in Pancreatic Islet- and Beta-Cell Area That Is Not Observed in Female Mice

At 12 weeks of age, male Ins1*^Cre/+^*;Rosa26-eYFP mice had larger islets (*p* < 0.01) as well as increased beta- and alpha-cell areas (*p* < 0.05 and *p* < 0.01, respectively) when compared to age-matched female mice (Figure 1A–C). Islet number was also increased (*p* < 0.05) in younger male mice, but the number of islet cells positive for both insulin and glucagon was decreased (*p* < 0.01) in 12-week-old males when compared to equivalent female mice (Figure 1D,E). Interestingly, at 52 weeks of age, female mice had increased (*p* < 0.05–0.001) islet-, alpha- and beta-cell areas, with a substantial augmentation in (*p* < 0.001) islet number, in comparison to equivalent males (Figure 1A–D). In further contrast to younger mice, at 52 weeks of age, male mice had a greater abundance (*p* < 0.05) of dual-hormone-positive islet cells than their female counterparts (Figure 1E). In keeping with this, there were prominent interactions between age and sex for islet area (F(1,20) = 15.43, *p* < 0.001), beta-cell area (F(1,20) = 11.05, *p* < 0.01), alpha-cell area (F(1,20) = 6.83, *p* < 0.05), islet number (F(1,20) = 22.55, *p* < 0.001) and dual-hormone-positive islet cells (F(1,20) = 8.05, *p* < 0.05).

### 2.3. Beta-Cell Proliferation Declines with Age, but Apoptosis Rates Exhibit Age- and Sex-Specific Differences

Beta-cell proliferation was reduced (*p* < 0.05) in young male mice when compared to females, with this sex-specific difference maintained (*p* < 0.001) at 52 weeks of age (Figure 2A). Interestingly, in terms of beta-cell apoptosis, rates were decreased (*p* < 0.01) in 12-week-old male mice but considerably increased (*p* < 0.001) in 52-week-old male mice when compared to respective age-matched females (Figure 3A). In general, beta-cell proliferation decreased with age in both sexes (Figure 2A), but beta-cell apoptosis declined with age in female mice and was amplified with age in male mice (Figure 3A). Alpha-cell proliferation and apoptosis rates were not different between sexes at 12 weeks of age (Figure 2B and Figure 3B). However, at 52 weeks of age, alpha-cell proliferation was increased (*p* < 0.05) in male mice, alongside a non-significant tendency for increased rates of apoptosis (Figure 2B and Figure 3B). Interestingly, there were significant interactions between age and sex for beta-cell apoptotic rates (F(1,20) = 17.32, *p* < 0.001), but not beta-cell proliferation (F(1,20) = 0.22, *p* = 0.65), alpha-cell proliferation (F(1,20) = 4.21, *p* = 0.06), or alpha-cell apoptosis (F(1,20) = 1.68, *p* = 0.2).

### 2.4. Islet Cell Plasticity, as Well as Beta-Cell Neogenesis, Exhibit Clear Sex-Specific Variations That Are Affected by Age

At 12 weeks of age, male Ins1*^Cre/+^*;Rosa26-eYFP mice exhibited reduced (*p* < 0.001) beta-cell dedifferentiation, together with increased (*p* < 0.05–0.001) beta-to-alpha-cell transdifferentiation as well as the association between ductal and beta-cell markers when compared to female mice of the same age (Figure 4A,B and Figure 5A,B). At 52 weeks of age, there was an almost complete reversal of these observations, with female mice exhibiting reduced (*p* < 0.01) beta-cell dedifferentiation, together with increased (*p* < 0.001) beta-to-alpha-cell transdifferentiation and endocrine-derived beta-cell neogenesis, when compared to respective males (Figure 4A,B and Figure 5A). The interactions between age and sex were significant for beta-cell dedifferentiation (F(1,20) = 39.17, *p* < 0.001) as well as beta-to-alpha-cell transdifferentiation (F(1,20) = 348.59, *p* < 0.001). The only parameter that was similar between sexes was CK19/insulin co-expression at 52 weeks of age (Figure 5B), but there was significant interaction between age and sex in relation to CK19/insulin co-expression (F(1,20) = 5.88, *p* < 0.05).

### 2.5. Observations in Glu^CreERT2^;ROSA26-eYFP Mice Corroborate Effects of Sex and Age on Islet Cell Plasticity in Ins1^Cre/+^;Rosa26-eYFP Mice

Alpha-cell dedifferentiation, defined as original GFP-positive alpha cells no longer staining positive for glucagon, was elevated in 12-week-old males (*p* < 0.001) but reduced (*p* < 0.05) in 52-week-old males, when compared to their age-matched female counterparts (Figure 6A). Accordingly, the interaction between age and sex was significant for alpha-cell dedifferentiation (F(1,20) = 43.19, *p* < 0.001). Similarly, alpha-to-beta-cell transdifferentiation was increased (*p* < 0.001) in male mice at 12 weeks but decreased (*p* < 0.01) at 52 weeks of age (Figure 6B), with significant interactions between age and sex for alpha-to-beta-cell transdifferentiation (F(1,20) = 8.23, *p* < 0.001). Notably, alpha-cell dedifferentiation, as well as the number of alpha-to-beta-cell transdifferentiation events, were elevated with age in female mice, but decreased with age in males (Figure 6A,B). Finally, the percentage of cells positive for glucagon but negative for GFP was similar in male and female Glu*^CreERT2^*;ROSA26-eYFP mice at 12 weeks but substantially elevated (*p* < 0.001) in males, relative to females, at 52 weeks (Figure 6C). Importantly, the interaction between age and sex was significant for the percentage of glucagon-positive, GFP-negative cells (F(1,20) = 7.65, *p* < 0.05).

## 3. Discussion

Although sex and ageing are well-recognised risk factors for impaired glucose tolerance and related metabolic disorders [19], there is limited information regarding potential differences in pancreatic islet architectural patterns between sexes and related subsequent adaptations to ageing. The novelty of this study lies in its pioneering exploration of islet cell plasticity in this context, a phenomenon now accepted as crucial for maintaining normal islet morphology and function [14]. We present original findings that changes in islet cell identity and lineage play a hitherto unappreciated role in regulating pancreatic islet morphology and consequently in the established sex- and age-induced disparities in beta-cell mass and metabolic control. Importantly, the current findings extend prior observations regarding the sex- and age-dependent nature of islet cell plasticity [15,18], highlighting that the ability of female mice to maintain beta-cell mass in ageing is associated with positive effects on islet cell lineage.

In keeping with studies in related Glu*^CreERT2^*;ROSA26-eYFP transgenic mice [18], young male Ins1*^Cre/+^*;Rosa26-eYFP mice possessed a greater number of larger pancreatic islets, alongside augmented alpha- and beta-cell areas, when compared to their female counterparts. In addition, as recently observed in Ins1*^Cre/+^*;Rosa26-eYFP male mice [15], there were subsequent age-related reductions of islet- and beta-cell areas in these animals. However, some debate still surrounds the impact of ageing on rodent pancreatic islet morphology, with reports of both a decline and augmentation of beta-cell mass with age [20,21,22]. Intriguingly, in contrast to males, female Ins1*^Cre/+^*;Rosa26-eYFP mice presented with enhanced islet- and beta-cell area at 52 weeks of age when compared with their respective 12-week-old controls. Whilst a body weight increase might represent one explanation [23], relative changes in body weight in both male and female mice were equivalent with age. Thus, this sex-based difference may be related to the positive impact of female sex hormones on islet cell survival over time [24], resulting in elevated blood glucose levels in male, but not female, mice at 52 weeks of age. While recent studies employing continuous glucose monitoring hint towards a lack of glycaemic variation between male and female mice [25], it is essential to note that this work was conducted in young healthy mice not exposed to islet stressors. Moreover, in humans, type 2 diabetes is generally more prevalent in adult men than in women [4], consistent with our observations. Whilst it would also be interesting to assess circulating insulin, triglycerides and free fatty acids in the groups of mice, the primary focus of the current study was assessment of pancreatic islet morphology and beta-cell plasticity.

It has been established that rates of beta-cell proliferation diminish with age in both humans and rodents [26,27], in good agreement with our findings in male and female Ins1*^Cre/+^*;Rosa26-eYFP mice. Of note, both young and ageing female mice had pronounced elevations of beta-cell proliferation when compared to males, alongside substantial reductions in apoptotic rates at 52 weeks of age. Clearly, these changes must be associated, in some part, with the augmented beta-cell mass in older female mice, possibly aligning to benefits of oestrogen on cell survival [28]. That said, rates of islet cell turnover were relatively elevated in the current study, which may impact overall interpretation. In contrast, increased beta-cell mass in younger male mice appears to be more related to derivation of new progenitor beta cells from a ductal source [29], together with reduced apoptotic rates of surviving beta cells. Notably, the association between ductal and beta-cell markers was not altered with age in female mice, suggesting that this pathway is less important for preservation of islet architecture over time. In support of this, others have also suggested that the contribution of duct cells towards normal beta cells and functional islets is minimal [30]. Intriguingly, sex-specific variations in pancreatic islet morphology could potentially modulate the insulo–acinar axis, where islet hormone gradients are known to influence surrounding exocrine tissue [31]. Such structural changes could alter the regulation of peri-insular acinar cell function and enzyme secretion profiles.

In terms of islet cell lineage, our preliminary observations of sex- and age-related variations in the number of islet cells staining positive for both insulin and glucagon provided good initial evidence for a role of islet cell plasticity in regulating overall pancreatic islet morphology. Accordingly, at 12 weeks of age, male Ins1*^Cre/+^*;Rosa26-eYFP mice presented with reduced beta-cell dedifferentiation when compared to females, yet beta-to-alpha-cell transdifferentiation was augmented despite reductions in dual-hormone-positively stained islet cells. Together, this would imply that preservation of beta-cell identity is of more physiological significance than generation of new beta cells in young male mice [32]. Ultimately, single-cell RNA sequencing experiments may be needed to fully confirm changes in islet cell lineage by assessing the expression of key markers of beta- and alpha-cell identity [12], but this is well outside the scope of the current study. Furthermore, while cell-fate transitions via transdifferentiation are certain in our lineage-tracing models, in terms of dedifferentiation, islet cells can undergo acute degranulation and a loss of detectable hormone expression under very specific circumstances, while still retaining their baseline cellular identity [33]. In the female setting, there appears to be an almost opposing effect on islet cell plasticity and identity at 12 weeks of age. That said, beta-to-alpha-cell transdifferentiation was maintained at extremely low levels in younger female mice and is likely to have some relevance for conservation of beta-cell mass. However, under the metabolic stress of ageing [34], more emphasis is placed on adaptations of islet cell lineage in female mice, which becomes critical for maintaining and promoting islet- and beta-cell mass over time. In good agreement, recent observations from hepatocyte nuclear factor 4-α (HNF4α) knockout (KO) mice, a transcription factor linked to beta-cell function and development, reveal elevated stress-induced beta-cell loss with age in HNF4α KO male mice, with protective effects in females being partially linked to positive modulation of beta-cell plasticity [35].

Whether male mice exhaust such islet cell lineage alteration pathways in their early life remains to be answered, but increased alpha-cell proliferation alongside potentially elevated alpha-cell neogenesis suggests an ultimately ineffective bid to expand the beta-cell progenitor pool in male mice [15]. This possible amplification of neogenesis was specifically determined through the detection of increased glucagon-positive, GFP-negative cells in older male Glu*^CreERT2^*;ROSA26-eYFP mice, despite a reduction in alpha-cell area. In favour of this concept, the number of glucagon-positive, GFP-negative islet cells was just above detection limits in 52-week-old female mice. Moreover, whilst observations of alpha-to-beta-cell transdifferentiation in young male Glu*^CreERT2^*;ROSA26-eYFP mice did not precisely mirror changes in beta-cell lineage in respective Ins1*^Cre/+^*;Rosa26-eYFP male mice, they do highlight over-active islet cell plasticity that may then lead to exhaustion, with elevated alpha-cell dedifferentiation in this cohort of mice also supporting that concept. Overall, it appears that preserved pancreatic islet cell morphology and related metabolic advantages in the female sex [7] relate to a more appropriate adaptation of islet cell transitioning events in the face of stressors, such as ageing, likely driven by hormonal triggers. However, direct assessment of the secretory function of these lineage-altered islet cell populations would be valuable for establishing physiological relevance, but such analyses are currently limited by available methodology. In addition, despite the appropriateness of both our transgenic models for detecting lineage alterations, the current observations on islet cell plasticity were made only at 12 and 52 weeks of age. In this regard, using islet implantation into the anterior chamber of the eye (ACE) technology in mice [36] could permit a more detailed temporal evaluation of islet cell differentiation events. Additional limitations include incomplete expression of detectable GFP in alpha cells of Glu*^CreERT2^*;ROSA26-eYFP mice [37], although tamoxifen-induced activation of the alpha-cell Cre recombinase enzyme clearly does not affect normal islet cell transdifferentiation [38]. Further, whilst restricted beta-cell recombination events have been observed in older Glu*^CreERT2^*;ROSA26-eYFP mice, over 99% of GFP-positive cells were still of alpha-cell origin [39]. Finally, triple co-staining for insulin, glucagon and GFP would have aided overall interpretation of islet cell lineage events, but this was not possible in the current setting.

In conclusion, the present study demonstrates clear differences in pancreatic islet morphology across sexes and a subsequent altered ability to adapt to age-related metabolic stress. Reduced beta-cell proliferation with age occurs independent of sex, but there are sex-specific differences in the survival of existing beta cells that support conservation of beta-cell mass in the female setting. Furthermore, middle-aged female mice retain the ability to effectively modify islet cell plasticity towards pathways that can sustain or expand the beta-cell pool, a response that is heavily compromised in ageing male mice. Thus, strategies that can enhance positive islet cell transitioning events may find therapeutic application for age-related decline in metabolic control.

## 4. Materials and Methods

### 4.1. Animals

Virgin male and female Ins1*^Cre/+^*;Rosa26-eYFP transgenic mice on a C57Bl/6 genetic background were bred in-house within the Biomedical and Behavioural Research Unit at Ulster University, Coleraine, UK and used at either 12 or 52 weeks of age. The origin and characteristics of these mice have been described previously [40,41]. Experimental mice were derived from different litters within our in-house breeding colony, minimising litter-specific bias and ensuring our findings are more reproducible. In relation to this, a small subset of the male Ins1*^Cre/+^*;Rosa26-eYFP mice used in the current study were littermates from our recent report on the impact of age on islet cell plasticity [15]. Observations were also made in age-matched male and female Glu*^CreERT2^*;ROSA26-eYFP transgenic mice, also on a C57Bl/6 genetic background. Unlike Ins1*^Cre/+^*;Rosa26-eYFP mice that have a constitutively active transgene, Glu*^CreERT2^*;ROSA26-eYFP mice express a tamoxifen-inducible (7 mg/mouse dissolved in corn oil, administered as a single intraperitoneal injection 3 days prior to culling) eYFP reporter specifically in cells expressing the glucagon gene [39]. All mice were housed in a temperature-controlled environment (22 ± 2 °C) on a 12 h light/dark cycle. They were maintained on standard rodent maintenance chow (Trouw Nutrition, Norwich, UK) and normal drinking water ad libitum. All experiments were approved by the Ulster University Animal Ethics Review Committee, conducted in accordance with the UK Animals (Scientific Procedures) Act 1986, and reported in line with the ARRIVE (Animal Research: Reporting of In Vivo Experiments) guidelines.

### 4.2. Immunohistochemistry

Mice were euthanised at either 12 or 52 weeks of age by lethal inhalation of CO_2_ followed by cervical dislocation, with body weight and blood glucose determined just prior to termination. Blood glucose was measured using a handheld Ascencia Contour blood glucose meter (Bayer Healthcare, Newbury, UK). Pancreatic tissue was excised and fixed in 4% paraformaldehyde for 48 h. Following fixation, tissue samples were dehydrated and cleared before embedding in paraffin and sectioning (5 µm) for immunohistochemistry analysis, as described previously [41]. Sections were picked at an interval of 1 every 10 sections, with a minimum of 10 islets examined per mouse. Briefly, slides were immersed in xylene to remove wax and rehydrated in a series of ethanol washes of reducing concentration (100–50%) followed by phosphate-buffered saline (PBS). Antigen retrieval was achieved by using a heated citrate buffer (90 °C, pH 6) for 20 min, followed by blocking in 4% bovine serum albumin (BSA). As reported previously, these fixation procedures quenched endogenous GFP fluorescence [42], necessitating use of a GFP primary antibody as detailed below. Primary antibodies, including insulin (1/400; Abcam ab6995, Abcam, Cambridge, UK), glucagon (1/1000; Abcam ab92517), GFP (1/500; Abcam ab5450), Ki-67 (1/500; Abcam ab15580) and CK-19 (1/500; Abcam ab76539) were then added, followed by appropriate Alexa Fluor secondary antibodies (all 1:400; Alexa Fluor 488 for green or 594 for red). For alpha-cell Ki-67 analyses, the highly specific polyclonal guinea pig anti-glucagon (1/200; PCA2/4:400 raised in-house) antibody was used [43]. Specifically, the following secondary antibodies were employed: donkey anti-goat Alexa Fluor 488 (Invitrogen, Paisley, UK, A-11055), donkey anti-mouse Alexa Fluor 594 (Invitrogen, A-21203), donkey anti-rabbit Alexa Fluor 594 (Invitrogen, A-21207), goat anti-rabbit Alexa Fluor 488 (Invitrogen, A-11008), goat anti-rabbit Alexa Fluor 594 (Invitrogen, A-11012), as well as goat anti-guinea pig Alexa Fluor 488 (Invitrogen, A-11073). Lastly, slides were exposed to DAPI to identify nuclei before washing in PBS and mounting with glass coverslips. For assessing apoptosis, commercially available TUNEL staining (Roche Diagnostics, Burgess Hill, UK) was carried out following the manufacturer’s guidance. Stained slides were imaged on an Olympus BX-51 fluorescent microscope using a 40X oil immersion objective fitted with DAPI (350 nm), TRITC (594 nm) and FITC (488 nm) filters.

### 4.3. Image Analysis

Cell^F^ imaging software (Olympus Soft Imaging Solutions, Münster, Germany, version 1.17) was employed to assess islet morphology as well as cellular proliferation, apoptosis, dedifferentiation, transdifferentiation and neogenesis, with blinded analysis of all images. Slides stained for insulin/glucagon were used to assess general pancreatic islet morphology and quantified on ImageJ (version 1.54p) software using a “closed polygon” tool to manually select the Region of Interest, with results expressed as total islet-, beta- and alpha-cell areas in μm^2^ [44]. Total islet area was considered as the complete area of insulin- and glucagon-positive stained cells. The number of islets per mm^2^ was quantified by counting islets in ten non-overlapping fields of view (10×) per section and expressed per total tissue area in each image. Slides stained with TUNEL or Ki-67 were used to assess apoptosis and proliferation, respectively, in either insulin- or glucagon-positive cells. Similarly, the association between ductal- and beta-cell markers was quantified by assessing the percentage of CK-19-positively stained pancreatic ductal cells co-expressing insulin. Lack of endogenous GFP fluorescence meant that spectral overlap was not an issue for these analyses. For islet endocrine lineage analysis, in transgenic Ins1*^Cre/+^*;Rosa26-eYFP mice, GFP-positive cells are always of initial beta-cell lineage. Thus, islet cells expressing both insulin and GFP are considered original beta cells, whereas cells positive for GFP but lacking insulin are dedifferentiated beta cells, whilst islet cells expressing insulin without GFP are regarded as new beta cells from a non-beta-cell source. Similarly, in Glu*^CreERT2^*;ROSA26-eYFP mice, GFP-positive cells are always of initial alpha-cell lineage. Therefore, for the current study, islet cells expressing both glucagon and GFP are deemed as original alpha cells in this model, whereas cells positive for GFP and insulin are new beta cells from an alpha-cell source. For each parameter assessed, 10–15 islets were examined per animal with at least 60 islets analysed per treatment group (*n* = 6 animals per group).

### 4.4. Statistical Analyses

Results were analysed using GraphPad Prism (version 8), with data presented as mean ± SEM. Comparative analyses between the groups of mice were carried out using a two-way analysis of variance (ANOVA). While all graphs display individual islet values to illustrate biological distribution, individual animals were defined as the experimental unit, and statistical significance was based strictly on the number of mice per group (*n* = 6 animals per group). When no significant interaction between age and sex was detected, the main effects of each factor were evaluated. For any significant main effects, pair-wise comparisons were performed using Bonferroni’s post hoc test. Results were deemed significant at *p* < 0.05.

## Figures and Tables

**Figure 1:**
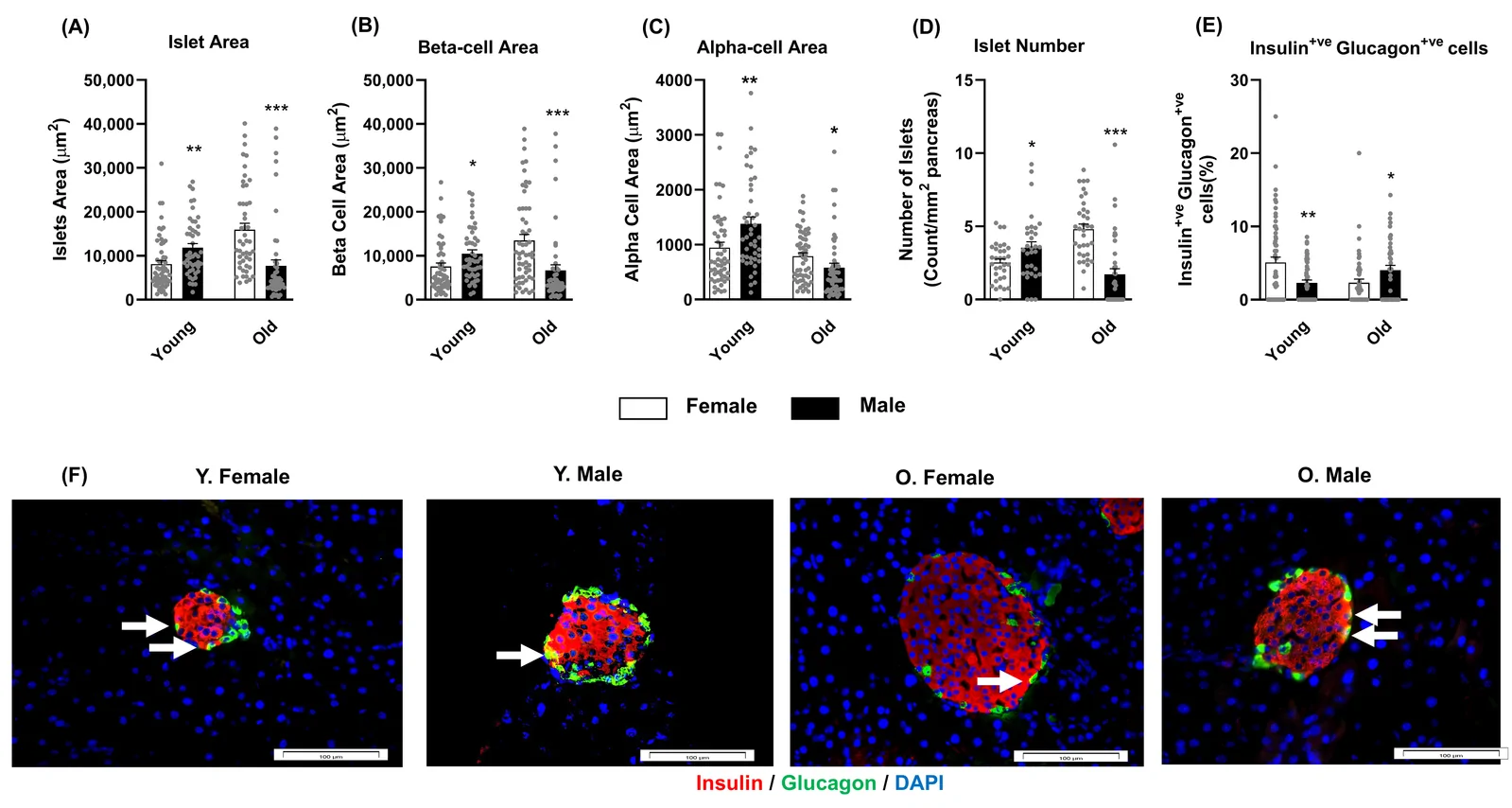
Pancreatic islet cell morphology in 12- and 52-week-old male and female Ins1*^Cre/+^*;Rosa26-eYFP mice. Immunohistochemistry was conducted to assess (**A**) islet area, (**B**) beta-cell area, (**C**) alpha-cell area, (**D**) islet number and (**E**) double-hormone-positive islet cells. (**F**) Representative islet images from all groups of mice showing insulin (red), glucagon (green) and DAPI (blue), with arrows depicting double-hormone-positive cells. Values are mean ± SEM (60 islets from *n* = 6 animals per group; at least 10 islets per mouse). * *p* < 0.05, ** *p* < 0.01 and *** *p* < 0.001 compared to respective 12- or 52-week-old female mice.

**Figure 2:**
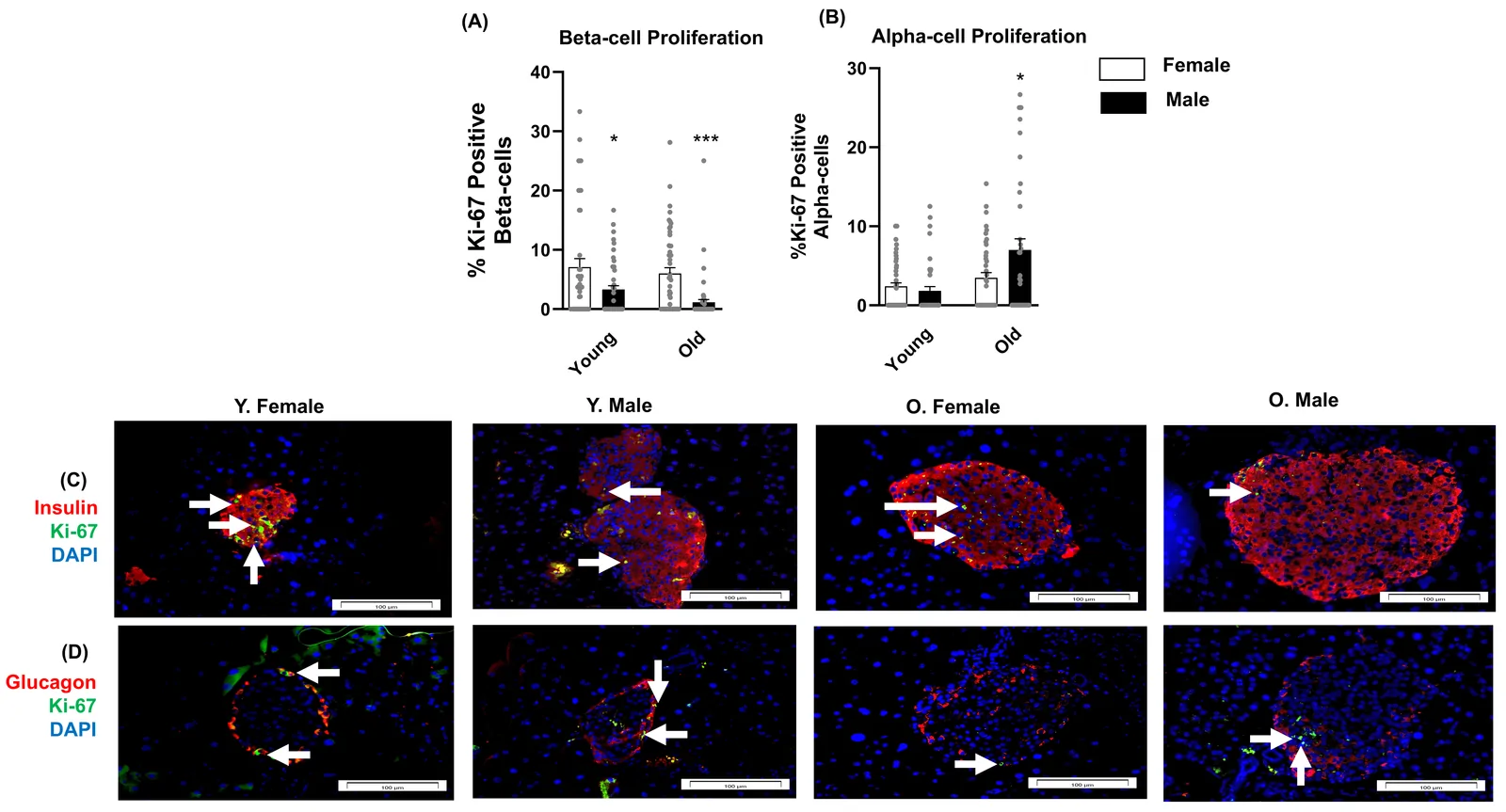
Islet alpha- and beta-cell proliferation in 12- and 52-week-old male and female Ins1*^Cre/+^*;Rosa26-eYFP mice. Immunohistochemistry was conducted to assess (**A**) beta-cell and (**B**) alpha-cell proliferation. (**C**,**D**) Representative images showing DAPI (blue) and Ki-67 (green) staining, with arrows highlighting positive co-staining alongside (**C**) insulin or (**D**) glucagon (red), as appropriate. Values are mean ± SEM (60 islets from *n* = 6 animals per group; at least 10 islets per mouse). * *p* < 0.05 and *** *p* < 0.001 compared to respective 12- or 52-week-old female mice.

**Figure 3:**
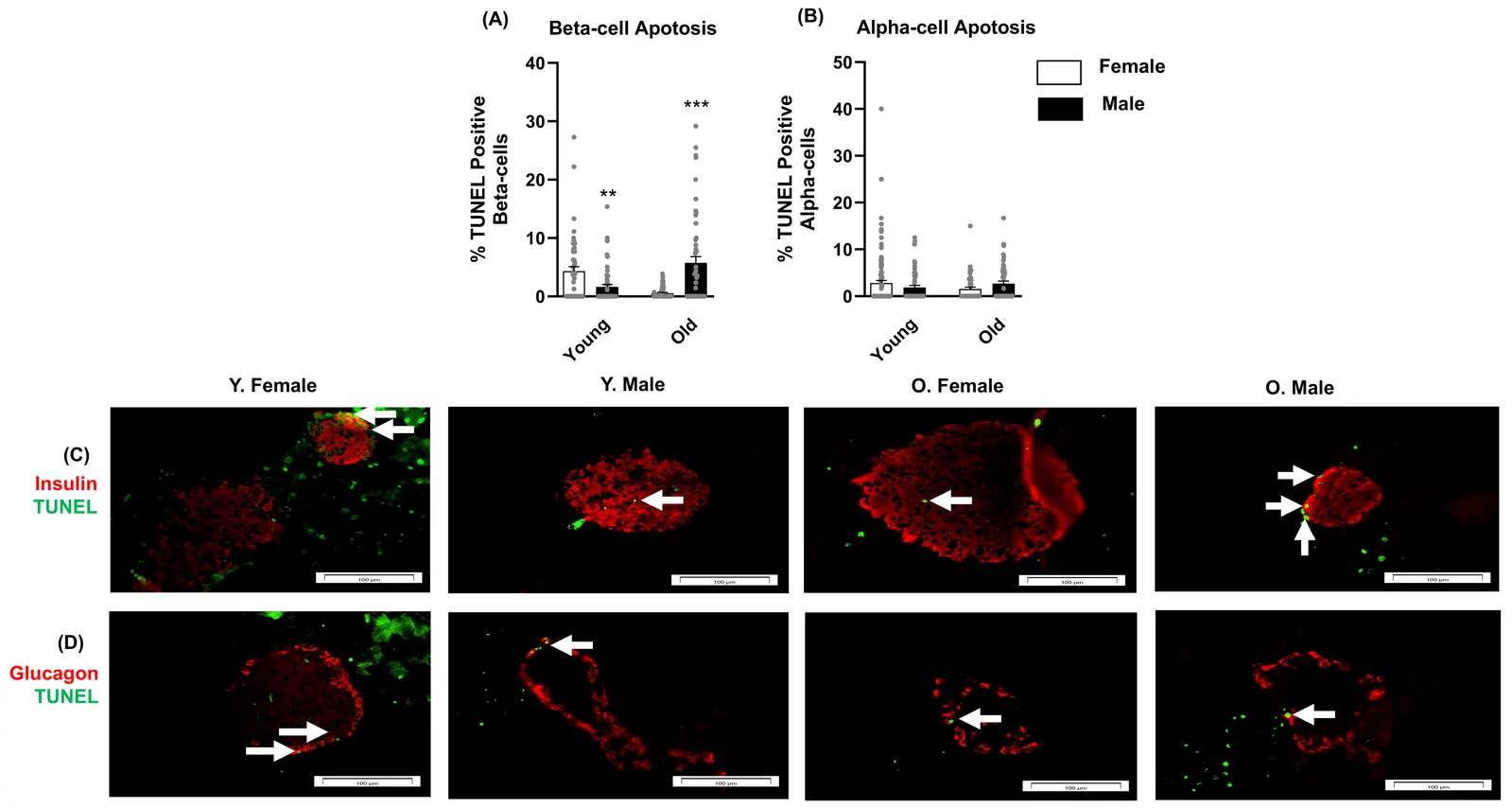
Islet alpha- and beta-cell apoptosis in 12- and 52-week-old male and female Ins1*^Cre/+^*;Rosa26-eYFP mice. Immunohistochemistry was conducted to assess (**A**) beta-cell and (**B**) alpha-cell apoptosis. (**C**,**D**) Representative images showing TUNEL (green) staining, with arrows highlighting positive co-staining alongside (**C**) insulin or (**D**) glucagon (red), as appropriate. Values are mean ± SEM (60 islets from *n* = 6 animals per group; at least 10 islets per mouse). ** *p* < 0.01 and *** *p* < 0.001 compared to respective 12- or 52-week-old female mice.

**Figure 4:**
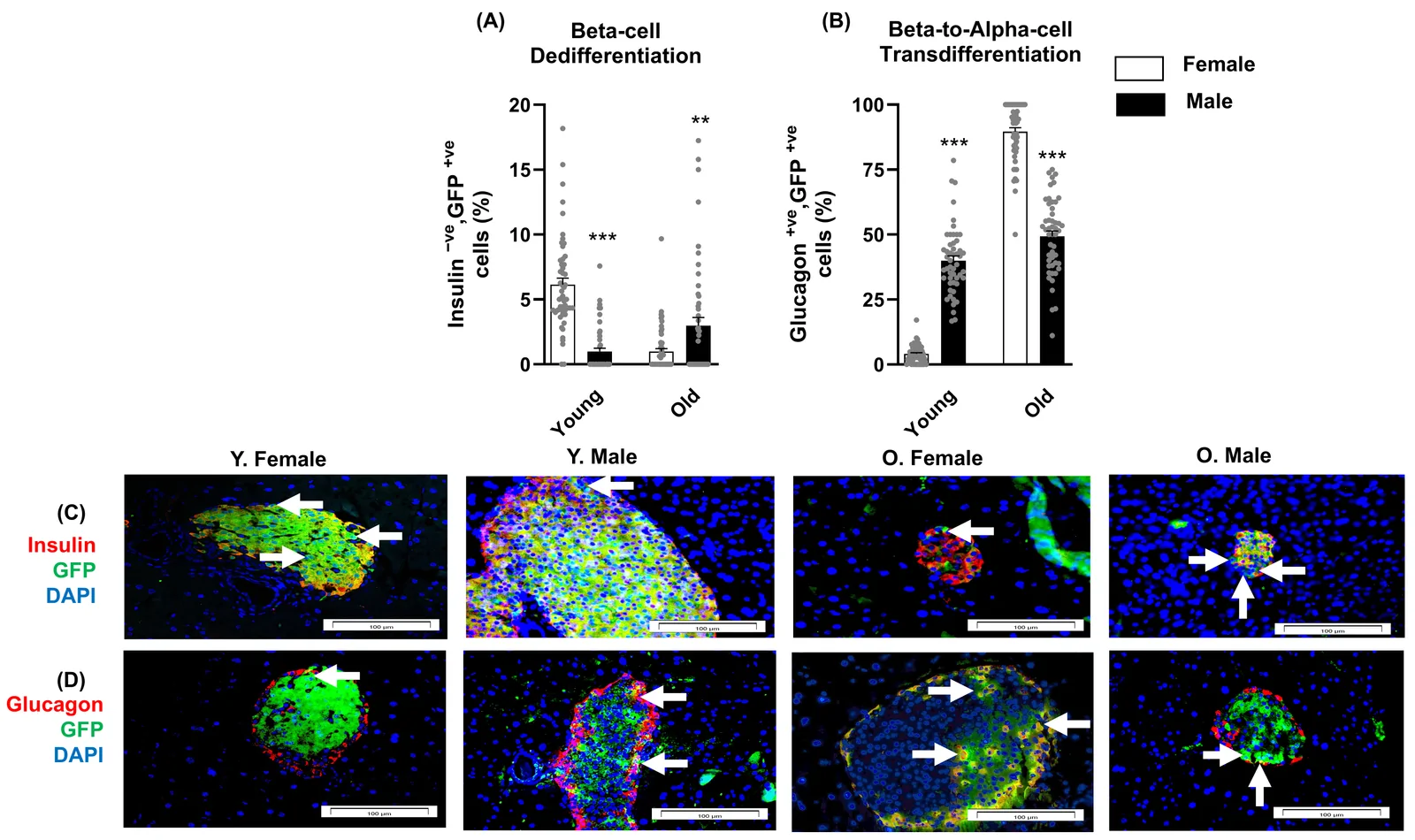
Beta-cell dedifferentiation and beta-to-alpha-cell transdifferentiation in 12- and 52-week-old male and female Ins1*^Cre/+^*;Rosa26-eYFP mice. Immunohistochemistry was conducted to assess (**A**) beta-cell dedifferentiation and (**B**) beta-to-alpha-cell transdifferentiation. (**C**,**D**) Representative images from each group showing (**C**) insulin (red), GFP (green) and DAPI (blue) as well as (**D**) glucagon (red), GFP (green) and DAPI (blue), with arrows highlighting positive co-staining, as appropriate. Values are mean ± SEM (60 islets from *n* = 6 animals per group; at least 10 islets per mouse). ** *p* < 0.01 and *** *p* < 0.001 compared to respective 12- or 52-week-old female mice.

**Figure 5:**
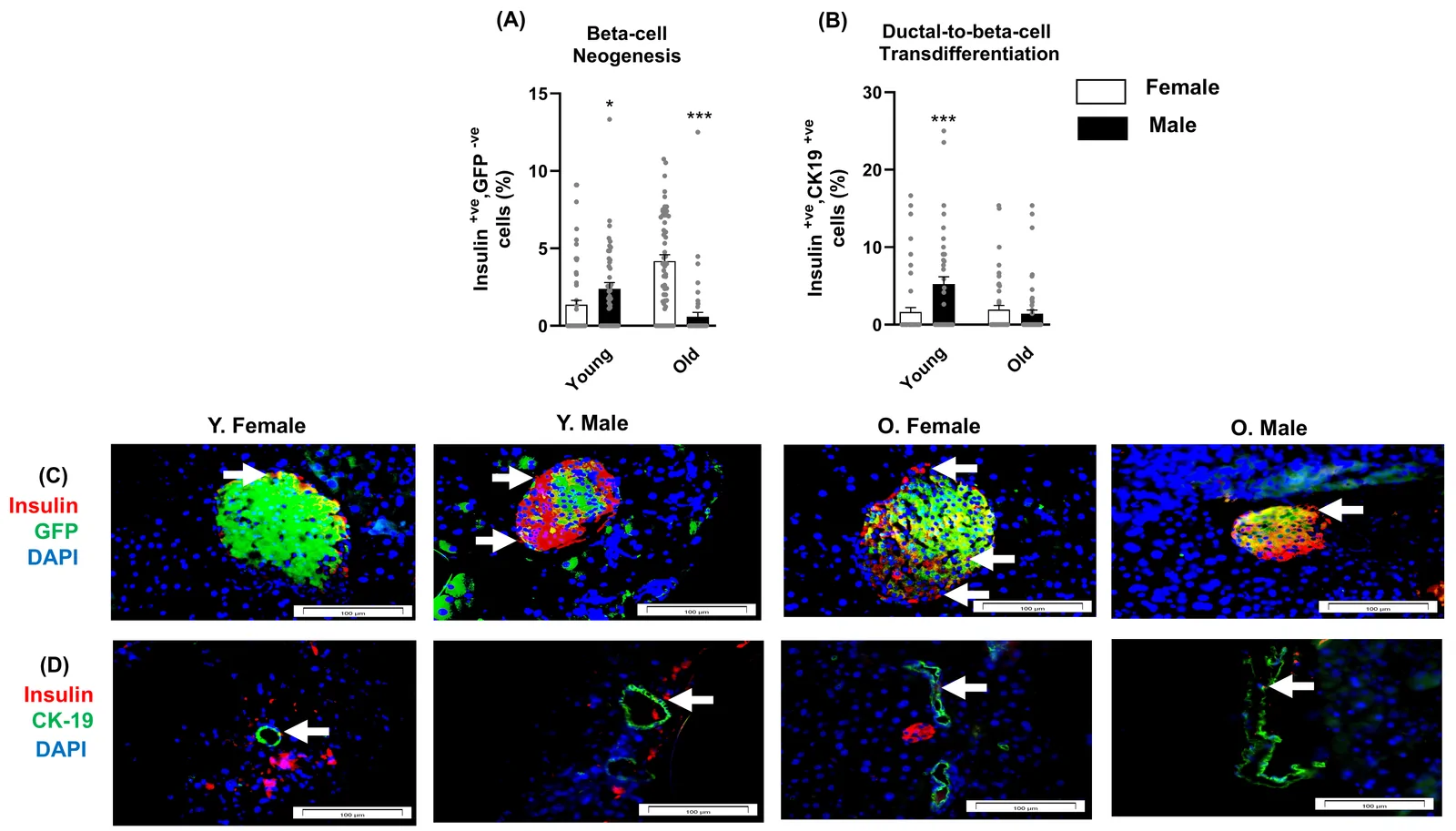
Beta-cell neogenesis and association between ductal and beta-cell markers in 12- and 52-week-old male and female Ins1*^Cre/+^*;Rosa26-eYFP mice. Immunohistochemistry was conducted to assess (**A**) beta-cell neogenesis and (**B**) potential ductal-to-beta-cell transdifferentiation. (**C**,**D**) Representative images from each group showing (**C**) insulin (red), GFP (green) and DAPI (blue) as well as (**D**) insulin (red), CK-19 (green) and DAPI (blue), with arrows highlighting positive co-staining, as appropriate. Values are mean ± SEM (60 islets from *n* = 6 animals per group; at least 10 islets per mouse). * *p* < 0.05 and *** *p* < 0.001 compared to respective 12- or 52-week-old female mice.

**Figure 6:**
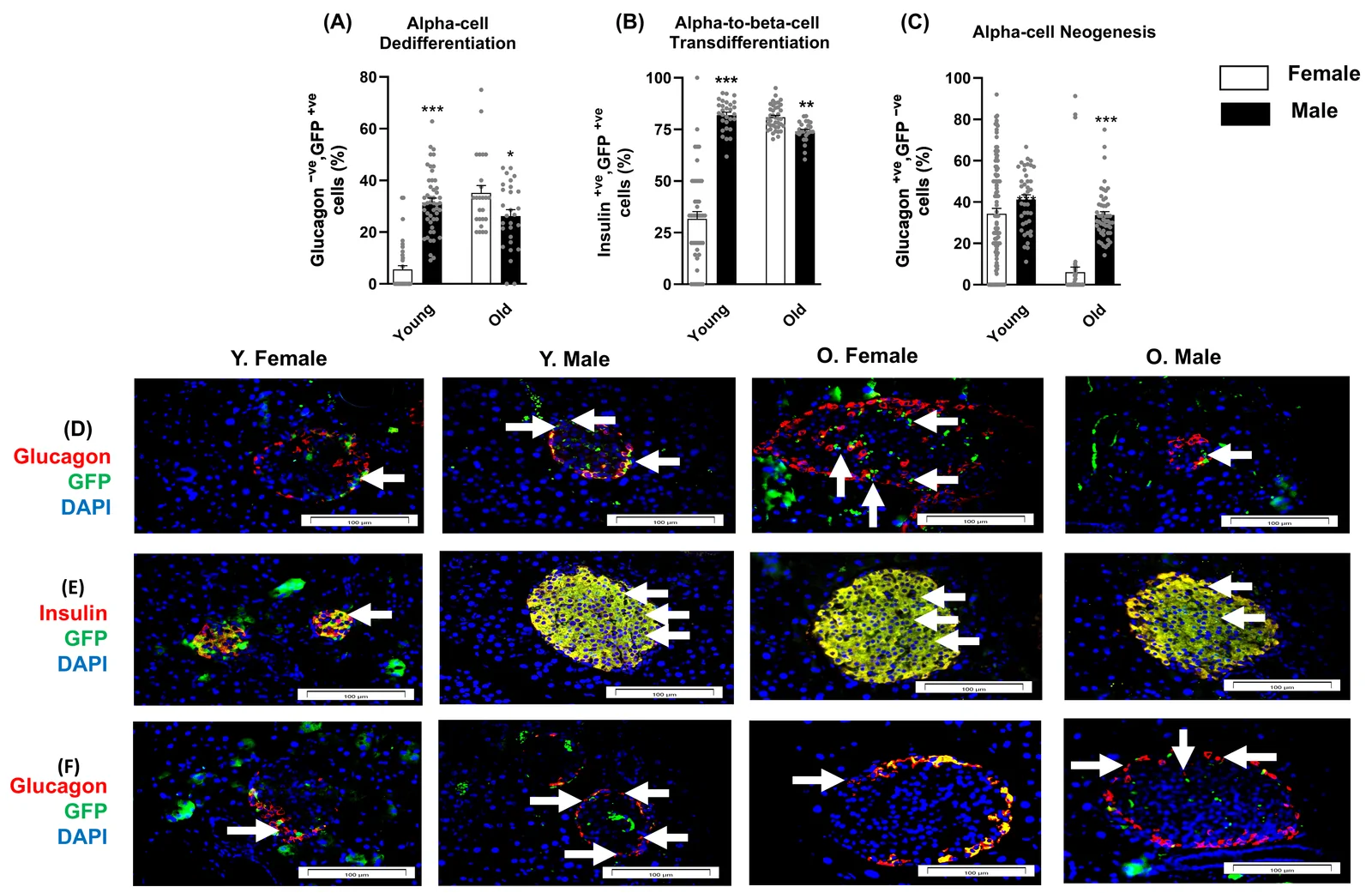
Alpha-cell dedifferentiation, alpha-to-beta-cell transdifferentiation and percentage of glucagon-positive, GFP-negative islet cells in 12- and 52-week-old male and female Glu*^CreERT2^*;ROSA26-eYFP mice. Immunohistochemistry was conducted to assess (**A**) alpha-cell dedifferentiation, (**B**) alpha-to-beta-cell transdifferentiation and (**C**) glucagon-positive, GFP-negative cells. (**D**–**F**) Representative images from each group showing (**D**,**F**) glucagon (red), GFP (green) and DAPI (blue), as well as (**E**) insulin (red), GFP (green) and DAPI (blue), with arrows highlighting positive co-staining, as appropriate. Values are mean ± SEM (60 islets from *n* = 6 animals per group; at least 10 islets per mouse). * *p* < 0.05, ** *p* < 0.01 and *** *p* < 0.001 compared to respective 12- or 52-week-old female mice.

**Table 1:** Body weight and blood glucose levels of 12- and 52-week-old male and female Ins1*^Cre/+^*;Rosa26-eYFP transgenic mice. Values are mean ± SEM (*n* = 6 animals). ** *p* < 0.01 compared to respective 12- or 52-week-old female mice.

	Body Weight (g)	Blood Glucose (mmol/L)
12-week-old female mice	22.0 ± 0.2	7.3 ± 0.3
12-week-old male mice	28.2 ± 1.6 **	7.0 ± 0.3
52-week-old female mice	33.3 ± 1.5	7.2 ± 0.2
52-week-old male mice	45.2 ± 3.1 **	8.8 ± 1.5 **

## Data Availability

The original contributions presented in this study are included in the article. Further inquiries can be directed to the corresponding author.

## References

[1] Allegra S., Chiara F., Di Grazia D., Gaspari M., De Francia S. (2023). Evaluation of Sex Differences in Preclinical Pharmacology Research: How Far Is Left to Go?. Pharmaceuticals.

[2] Corona G., Isidori A.M., Aversa A., Bonomi M., Ferlin A., Foresta C., La Vignera S., Maggi M., Pivonello R., Vignozzi L. (2020). Male and female sexual dysfunction in diabetic subjects: Focus on new antihyperglycemic drugs. Rev. Endocr. Metab. Disord..

[3] Arioglu-Inan E., Kayki-Mutlu G. (2023). Sex differences in glucose homeostasis. Handb. Exp. Pharmacol..

[4] Kautzky-Willer A., Leutner M., Harreiter J. (2023). Sex differences in type 2 diabetes. Diabetologia.

[5] Hanlon P., Butterly E., Wei L., Wightman H., Almazam S.A.M., Alsallumi K., Crowther J., McChrystal R., Rennison H., Hughes K. (2025). Age and Sex Differences in Efficacy of Treatments for Type 2 Diabetes: A Network Meta-Analysis. JAMA.

[6] Wrublewsky S., Widmann A.V., Bickelmann C., Rafacho A., Roma L.P., Laschke M.W., Ampofo E. (2025). Sex-Specific Differences in the Revascularization of Grafted Pancreatic Islets. Cells.

[7] Gannon M., Kulkarni R.N., Hubert M.T., Mauvais-Jarvis F. (2018). Sex differences underlying pancreatic islet biology and its dysfunction. Mol. Metab..

[8] Zhou Z., Ribas V., Rajbhandari P., Drew B.G., Moore T.M., Fluitt A.H., Reddish B.R., Whitney K.A., Georgia S., Vergnes L. (2018). Estrogen receptor α protects pancreatic β-cells from apoptosis by preserving mitochondrial function and suppressing endoplasmic reticulum stress. J. Biol. Chem..

[9] Xu W., Morford J., Mauvais-Jarvis F. (2019). Emerging role of testosterone in pancreatic β cell function and insulin secretion. J. Endocrinol..

[10] Şahin B., Soyer C., Çelik S., Ulubaşoğlu H., Sel G. (2020). The impact of the type of menopause and menopausal duration on the development of Pre-diabetes Mellitus and diabetes Mellitus in postmenopausal women. Turk. J. Diabetes Obes..

[11] Bailey C.J., Flatt P.R. (1982). Hormonal control of glucose homeostasis during development and ageing in mice. Metabolism.

[12] Talchai C., Xuan S., Lin H.V., Sussel L., Accili D. (2012). Pancreatic β cell dedifferentiation as a mechanism of diabetic β cell failure. Cell.

[13] Meier J.J., Butler A.E., Saisho Y., Monchamp T., Galasso R., Bhushan A., Rizza R.A., Butler P.C. (2008). β-cell replication is the primary mechanism subserving the postnatal expansion of β-cell mass in humans. Diabetes.

[14] Tanday N., Tarasov A.I., Moffett R.C., Flatt P.R., Irwin N. (2024). Pancreatic islet cell plasticity: Pathogenic or therapeutically exploitable?. Diabetes Obes. Metab..

[15] Dubey V., Tanday N., Sridhar A., Tarasov A.I., Flatt P.R., Moffett R.C., Irwin N. (2026). Adverse effect of age on pancreatic islet morphology, islet cell turnover and lineage-labelled hormone expression in mice. J. Endocrinol. Investig..

[16] Tanday N., Irwin N., Flatt P.R., Moffett R.C. (2020). Dapagliflozin exerts positive effects on beta cells, decreases glucagon and does not alter beta- to alpha-cell transdifferentiation in mouse models of diabetes and insulin resistance. Biochem. Pharmacol..

[17] Tanday N., Irwin N., Moffett R.C., Flatt P.R., O’Harte F.P.M. (2020). Beneficial actions of a long-acting apelin analogue in diabetes are related to positive effects on islet cell turnover and transdifferentiation. Diabetes Obes. Metab..

[18] Tanday N., Coulter-Parkhill A., Moffett R.C., Suruli K., Dubey V., Flatt P.R., Irwin N. (2023). Sex-based impact of pancreatic islet stressors in GluCreERT2/Rosa26-eYFP mice. J. Endocrinol..

[19] Tura A., Pacini G., Moro E., Vrbíková J., Bendlová B., Kautzky-Willer A. (2018). Sex- and age-related differences of metabolic parameters in impaired glucose metabolism and type 2 diabetes compared to normal glucose tolerance. Diabetes Res. Clin. Pract..

[20] Kawasaki F., Matsuda M., Kanda Y., Inoue H., Kaku K. (2005). Structural and functional analysis of pancreatic islets preserved by pioglitazone in *db/db* mice. Am. J. Physiol. Metab..

[21] Tudurí E., Soriano S., Almagro L., García-Heredia A., Rafacho A., Alonso-Magdalena P., Nadal Á., Quesada I. (2022). The effects of aging on male mouse pancreatic β-cell function involve multiple events in the regulation of secretion: Influence of insulin sensitivity. J. Gerontol. Ser. A Biol. Sci. Med. Sci..

[22] Tudurí E., Soriano S., Almagro L., Montanya E., Alonso-Magdalena P., Nadal Á., Quesada I. (2022). The pancreatic β-cell in ageing: Implications in age-related diabetes. Ageing Res. Rev..

[23] Saisho Y., Butler A.E., Manesso E., Elashoff D., Rizza R.A., Butler P.C. (2013). β-cell mass and turnover in humans: Effects of obesity and aging. Diabetes Care.

[24] Tiano J.P., Mauvais-Jarvis F. (2012). Importance of oestrogen receptors to preserve functional β-cell mass in diabetes. Nat. Rev. Endocrinol..

[25] Kennard M., Gatward L.F.D., Nandi M., King A.J.F. (2026). Female Mice Are Not More Variable Than Males: Evidence from continuous glucose monitoring in normoglycaemic C57BL/6 mice. Diabetes Obes. Metab..

[26] Rankin M.M., Kushner J.A. (2009). Adaptive β-cell proliferation is severely restricted with advanced age. Diabetes.

[27] Helman A., Avrahami D., Klochendler A., Glaser B., Kaestner K.H., Ben-Porath I., Dor Y. (2016). Effects of ageing and senescence on pancreatic β-cell function. Diabetes Obes. Metab..

[28] Le May C., Chu K., Hu M., Ortega C.S., Simpson E.R., Korach K.S., Tsai M.-J., Mauvais-Jarvis F. (2006). Estrogens protect pancreatic β-cells from apoptosis and prevent insulin-deficient diabetes mellitus in mice. Proc. Natl. Acad. Sci. USA.

[29] Rosenberg L. (1995). In vivo cell transformation: Neogenesis of beta cells from pancreatic ductal cells. Cell Transplant..

[30] Liu Q., Jiang Y., Zhu L., Qian J., Wang C., Yang T., Prasadan K., Gittes G.K., Xiao X. (2021). Insulin-positive ductal cells do not migrate into preexisting islets during pregnancy. Exp. Mol. Med..

[31] Pierzynowski S.G., Gregory P.C., Filip R., Woliński J., Pierzynowska K.G. (2018). Glucose homeostasis dependency on acini–islet–acinar (AIA) axis communication: A new possible pathophysiological hypothesis regarding diabetes mellitus. Nutr. Diabetes.

[32] Spijker H.S., Song H., Ellenbroek J.H., Roefs M.M., Engelse M.A., Bos E., Koster A.J., Rabelink T.J., Hansen B.C., Clark A. (2015). Loss of β-cell identity occurs in type 2 diabetes and is associated with islet amyloid deposits. Diabetes.

[33] Brereton M.F., Rohm M., Ashcroft F.M. (2016). β-Cell dysfunction in diabetes: A crisis of identity?. Diabetes Obes. Metab..

[34] Kautzky-Willer A., Brazzale A.R., Moro E., Vrbíková J., Bendlova B., Sbrignadello S., Tura A., Pacini G. (2012). Influence of increasing BMI on insulin sensitivity and secretion in normotolerant men and women of a wide age span. Obesity.

[35] Villaca C.B.P., Oliveira V.R., Santos G.J., Ortis F. (2025). The effect of HNF4α knockout in beta cells is age and sex dependent. Islets.

[36] Yang S.-N., Shi Y., Berggren P.-O. (2024). The anterior chamber of the eye technology and its anatomical, optical, and immunological bases. Physiol. Rev..

[37] Sarnobat D., Moffett R.C., Gault V.A., Tanday N., Reimann F., Gribble F.M., Flatt P.R., Irwin N. (2020). Effects of long-acting GIP, xenin and oxyntomodulin peptide analogues on alpha-cell transdifferentiation in insulin-deficient diabetic GluCreERT2;ROSA26-eYFP mice. Peptides.

[38] Thorel F., Népote V., Avril I., Kohno K., Desgraz R., Chera S., Herrera P.L. (2010). Conversion of adult pancreatic alpha-cells to beta-cells after extreme beta-cell loss. Nature.

[39] Campbell J.R., Martchenko A., Sweeney M.E., Maalouf M.F., Psichas A., Gribble F.M., Reimann F., Brubaker P.L. (2020). Essential Role of Syntaxin-Binding Protein-1 in the Regulation of Glucagon-like Peptide-1 Secretion. Endocrinology.

[40] Thorens B., Tarussio D., Maestro M.A., Rovira M., Heikkilä E., Ferrer J. (2015). Ins1(Cre) knock-in mice for beta cell-specific gene recombination. Diabetologia.

[41] Tanday N., Flatt P.R., Irwin N., Moffett R.C. (2020). Liraglutide and sitagliptin counter beta- to alpha-cell transdifferentiation in diabetes. J. Endocrinol..

[42] Zhanmu O., Zhao P., Yang Y., Yang X., Gong H., Li X. (2019). Maintenance of Fluorescence During Paraffin Embedding of Fluorescent Protein-Labeled Specimens. Front. Neurosci..

[43] Lafferty R.A., Tanday N., Moffett R.C., Reimann F., Gribble F.M., Flatt P.R., Irwin N. (2021). Positive Effects of NPY1 Receptor Activation on Islet Structure Are Driven by Pancreatic Alpha- and Beta-Cell Transdifferentiation in Diabetic Mice. Front. Endocrinol..

[44] Khan D., Vasu S., Moffett R.C., Irwin N., Flatt P.R. (2016). Islet distribution of Peptide YY and its regulatory role in primary mouse islets and immortalised rodent and human beta-cell function and survival. Mol. Cell. Endocrinol..

